# Relationship between dietary carbohydrate quality index and metabolic syndrome among type 2 diabetes mellitus subjects: a case-control study from Ghana

**DOI:** 10.1186/s12889-021-10593-3

**Published:** 2021-03-17

**Authors:** Sufyan Bakuri Suara, Fereydoun Siassi, Mahama Saaka, Abbas Rahimiforoushani, Gity Sotoudeh

**Affiliations:** 1 Department of Community Nutrition, School of Nutritional Sciences and Dietetics, International Campus, Tehran University of Medical Sciences, Number 21 Dameshgh St. Vali-e Asr Ave., Tehran, 1416753955 Iran; 2 Department of Community Nutrition, School of Nutritional Sciences and Dietetics, Tehran University of Medical Sciences, Hojatdost street, Naderi street, Keshavarz Blv, Tehran, Iran; 3 Department of Nutritional Sciences, School of Allied Health Sciences, University for Development Studies, Post Office Box 1350, Tamale, Ghana; 4 Department of Epidemiology and Biostatistics, School of Public Health, Tehran University of Medical Sciences, Tehran, Iran

**Keywords:** Metabolic syndrome, Carbohydrate quality index, Obesity, Dyslipidemia, Overweight

## Abstract

**Background:**

Dietary carbohydrate quality may play an important role in disease development. We evaluated the association between carbohydrate quality index (CQI) and the odds of metabolic syndrome (MetS) in type 2 diabetes mellitus (T2DM) subjects in Ghana.

**Methods:**

In this case-control study, we analyzed data using 124 T2DM subjects. We obtained dietary information using 2-day 24-h dietary recalls. We calculated CQI from dietary fiber, glycemic index, whole grains/total grains ratio, and solid carbohydrates/total carbohydrates ratio. Serum lipid profiles were measured after an overnight fast of 8–12 h.

**Results:**

Upon adjustments for the effects of covariates, the CQI showed a positive association with high-density lipoprotein cholesterol concentration (beta coefficient (β) = 0.24; standard error (SE) = 0.20; P for trend = 0.01), and an inverse relationship with waist circumference (β = − 17.29; SE = 4.00; P for trend < 0.001), systolic blood pressure (β = − 15.74; SE = 4.69; P for trend < 0.001), diastolic blood pressure (β = − 7.23; SE = 2.97; P for trend = 0.02), and triglyceride concentrations (β = − 0.43; SE = 0.11; P for trend < 0.001). Overall, the CQI had an inverse relationship with the odds of MetS (Odds ratio _tertile 3 vs.1_ 0.05; 95% Confidence interval: 0.01–0.23; p-trend < 0.001). Also, a positive correlation was found between the CQI and fiber, but the CQI showed a negative relationship with dietary glycemic index.

**Conclusions:**

The present results suggest an inverse association between the CQI of a diet and the odds of MetS. The CQI approach of dietary recommendation may be a useful strategy for dietary carbohydrate selection for the prevention of MetS.

## Background

In most countries, about 20 to 30% of the general population could be suffering from metabolic syndrome (MetS) [1, 2]. Among the Ghanaians with type 2 diabetes mellitus (T2DM), a high prevalence of MetS (24–78.8%) has been reported using different diagnostic criteria [3–7]. The MetS is associated with higher rates of mortality [8], cardiovascular disease (CVD) and cancers [9], and infertility in both men [10] and women [11].

Diet and lifestyle interventions may be more effective in the prevention of MetS development than pharmacological agents [12–15]. Moreover, lifestyle modifications such as increased physical activity, adherence to a healthy diet, and weight loss are said to be associated with the reversion of MetS and its risk components [16–22]. However, previous studies have mainly focused on investigating associations between limited indicators of dietary carbohydrate (CHO) quality [glycemic index (GI), glycemic load (GL), fiber content, sugar-sweetened beverage intake and whole-grain food consumption] and the odds of MetS amongst T2DM subjects [23–32].

The above previous approaches [23–32] of evaluating dietary CHO quality are narrow and may not be representative of a holistic metabolic capacity of CHO diets. Therefore, a more comprehensive assessment of dietary CHO quality may be a better alternative in investigating relationships between the quality of dietary CHO consumed and the odds of MetS in T2DM subjects. In line with this, a previous study defined CQI by taking into account dietary fiber intake; GI; ratio of CHO consumed from whole grains to CHO consumed from total grains (WGTG), and ratio of CHO consumed from solid food items to total CHO consumed (RSCTC) [33]. Although the CQI brings together several dimensions of dietary CHO quality and may be an effective tool for nutrition counseling, very few studies have examined its relationship with metabolic outcomes [34–36]. Obesity is considered an important risk for the development of MetS [37]. In the recent past, we reported an inverse association between the CQI and general and abdominal obesity in Ghanaian healthy women [34]. However, this was a healthy population. Therefore, investigating the association between CQI and MetS in T2DM may provide useful data for the prevention of MetS.

Previous studies among the T2DM subjects in Africa only focused on assessing the level of physical activity [38], and the barriers to adequate nutrition in Ghana [39, 40]. This perspective only provides limited insight into how to develop diet-related interventions that may be useful for the prevention of MetS, particularly amongst the T2DM subjects. Hence, a broader approach to the understanding of the quality of dietary CHO may be beneficial. Previously, Amugsi et al. [41] found that about 90% of the daily energy needs in the Ghanaian adult population come from dietary carbohydrates sources. The high intake of dietary CHO coupled with a high prevalence of MetS in the country could suggest that the intake of CHO may play a leading role in the health maintenance in the Ghanaian population than protein and fat intake. Hence, a broader evaluation of the quality of dietary CHO consumption amongst T2DM subjects may provide some information for the prevention of MetS and its risk components. Thus, the purpose of this study was to determine the association between dietary CQI and the odds of MetS among T2DM subjects in Tamale Metropolis, Ghana.

## Methods

### Study design and population

**Fig. 1:**
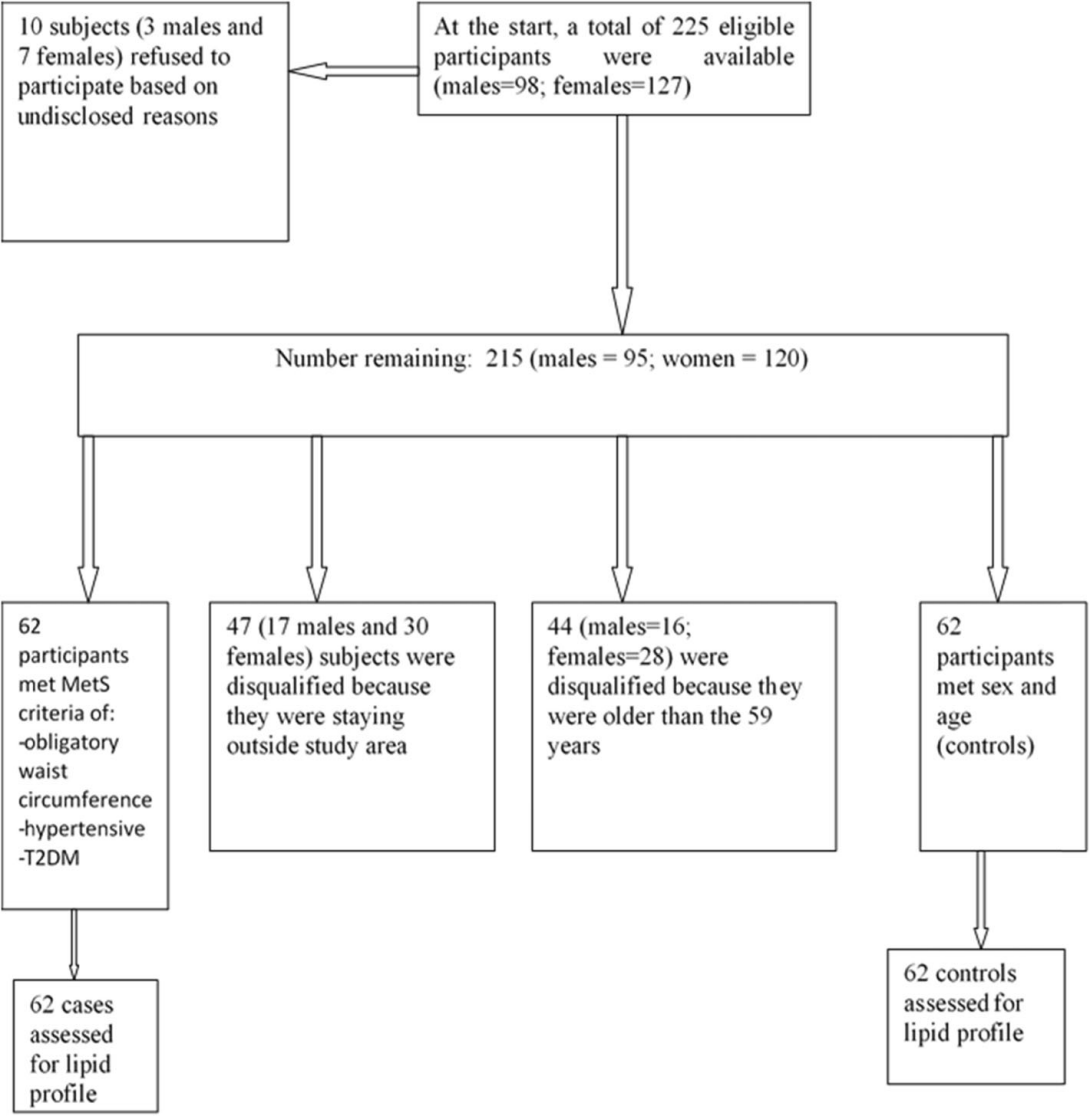
Study subjects’ sampling flow chart

This case-control study was performed using registered T2DM patients who attend to diabetic clinics for their routine medical care within Tamale Metropolis. Recruitment of participants (Fig. 1) and data collection span the period of April to July 2019. The T2DM patients were assessed and 124 participants were recruited [62 of patients had MetS (case group) and 62 subjects were without the MetS (control group)]. Overall, 44, 42, and 38 patients were recruited from Tamale Teaching Hospital, Tamale Central Hospital, and Tamale West Hospital, respectively. The MetS was defined according to the International Diabetes Federation (IDF) [42]. In the definition, MetS can be diagnosed if waist circumference (WC): ≥ 94 cm (Male), ≥ 80 cm (Female) is accompanied by any two (2) of the following four (4) factors: raised triglycerides (TG) [≥ 150 mg/dl (1.7 mmol/L) or specific treatment for this lipid abnormality]; reduced high density lipoprotein cholesterol (HDL-c) [< 40 mg/dl (1.03 mmol/L) in males, or < 50 mg/dl (1.29 mmol/L) in females or specific treatment for this lipid abnormality]; raised blood pressure [systolic blood pressure ≥ 130 or diastolic blood pressure ≥ 85 mmHg or treatment of previously diagnosed hypertension]; Raised fasting plasma glucose [(FPG) ≥ 100 mg/dl (5.6 mmol/L), or previously diagnosed T2DM] [42].

To participate in the study, the subjects must have lived in the Tamale Metropolis, Ghana, for at least a year prior to the study. Moreover, the participants should have been living with T2DM for at least 1 year. However, we excluded potential subjects with known diseases (renal disease, thyroid disease, confirmed malaria, and human immune deficiency virus). Furthermore, pregnant and lactating women were excluded. Also, potential **participants with** severe nausea and vomiting were disqualified from participating in the study. We also excluded current smokers, insulin users and those placed on special diet therapies. Additionally, cases and controls were obtained based on frequency matching for the age (18–39, 40–49, and 50–59 years) and sex.

The main objective of the study was to calculate the odds of MetS across a sex-specific energy-adjusted CQI tertiles. So, based on a previous study [24], we adopted the odds of 3.19 for MetS between two quintiles of GI (Q3, Q1) with Q1 as a reference, to determine the number of subjects that we recruited into the case and the control groups. We used an online scientific protocol for the sample size calculation [43]. Based on the protocol, 62 cases and 62 controls were sampled.

### Biochemical parameters

Serum TG, total cholesterol (TC), HDL-C, and low-density lipoprotein cholesterol (LDL-C) of diabetic patients were assayed to establish their metabolic profiles. The Tamale Teaching Hospital laboratory and Diabetic care clinics at Tamale Central Hospital and Tamale West Hospital were used as sentinel points for patient enrolment. Three milliliters of venous blood sample were taken from the antecubital vein of each subject using an aseptic technique. The Venepuncture was done by an experienced phlebotomist and the samples immediately transferred into serum separator gel tubes which were then placed in a test tube rack. The specimen was collected in the morning after an overnight fast of 8–12 h. Following clot retraction, the serum separator tubes were centrifuged at 3000 rpm for 10 min and the serum transferred into plain cryovials and frozen at − 20 °C and stored for 6 six weeks before they were analyzed. All assays were done using the Vital Scientific’s Flexor Junior Fully automated (with software version 4.1.x; manufacturer: Vital Scientific B. V, Netherlands, product ID: 6002–950–410-06) and Elitech reagents, calibrators, and controls manufactured by ElitechGroup, Puteaux, France. The assays were carried out at the Tamale Central Hospital Laboratory and in accordance with manufacturers’ recommended protocols. Standard quality assurance procedures as per the ISO15189 and laboratories policies were observed during the pre-analytical, analytical and post-analytical stages.

### Blood pressure (BP) measurement

The blood pressure values of T2DM patients were measured after 10 min of rest while subjects were in a supine position using a digital arm sphygmomanometer [(OMRON HEALTHCARE Co. Ltd., Japan; model: OMRON M6 AC (HEM-7322-E); measurement range: pressure: 0 to 299 mmHg; pulse: 40 to 180 beats/min; accuracy: pressure: ±3 mmHg; pulse: ±5% of display reading. Cuff circumference 22 to 42 cm)]. The left arm was used and it was ensured that the upper arm was bare and un-constricted by clothing, without the legs crossed and arm resting firmly and supported on a table. Readings were recorded to the nearest 0.5 mmHg.

### Patient and public involvement

This study included a sample of T2DM subjects. Although the participants were not involved in the study design process, their recruitment and participation were based on their own will.

### Dietary assessment and CQI calculation

All dietary data were collected using repeated, non-consecutive 2-day 24-h dietary recalls. We used real food items, food models, and kitchen weighing tools to guide study subjects in their estimation of food portion sizes consumed. A trained caterer was deployed to conduct face-to-face interviews following a 3-stage multiple-pass approach. This approach involves a quick listing of food items by respondents without any interruptions by the interviewer. This was followed by a detailed description and prompting for possibly forgotten food items. We obtained GI values from international tables [44]. Glucose was used as the reference (GI for glucose = 100). We used the mean of the GI values in scenarios where more than one eligible GI values were available for a given foodstuff. GI values assessed using healthy subjects were prioritized in our selection of reference GI values. We obtained the GI for millet porridge from the University of Sydney GI database [45]. Also, the GI of sorghum [46], tuo-zaafi, and banku [47] were obtained from published articles. The carbohydrate content of dietary intake was determined using standard portion sizes from the United States Department of Agriculture (USDA) food composition databases [48]. In the analysis, the mean daily values of dietary energy, fiber, and total carbohydrates from the 2-day 24-h recalls were used. The weighted daily dietary GIs were calculated following a standard protocol [35]: Weighted GI = ∑CHOi × GIi / daily total food carbohydrate content; where CHOi is each food’s carbohydrate content, GIi is each food’s GI. The GL value was calculated for each participant by multiplying the carbohydrate content in grams obtained from the portion of food consumed by the corresponding GI of that food divided by 100. To obtain the daily weighted GL, we summed up the individual GL values for each food [49]. By definition, liquid carbohydrates were calculated as the sum of the carbohydrates from all sugar-sweetened beverages and fruit juices consumed. Also, solid carbohydrates were considered to be the carbohydrate content of the rest of the carbohydrate-based food items [36].

The CQI was computed based on the energy-adjusted amount of total carbohydrate intake values using the residual method [50]. The CQI was defined by summing up the following four criteria: the ratio of solid carbohydrates to total carbohydrates, dietary fiber intake (g/day), GI, and the ratio of whole grains to total grains (whole grains, refined grains, and their products). For each of these four criteria, subjects were classified into quintile (Qs) and received a value (ranging from 1 to 5) according to each Q. However, we reversed the scoring of GI Qs; thus, those in the Q5 received 1 point and those in the Q1 received 5 points. Subsequently, the CQI was computed by adding together all the values of the four criteria (ranging from 4 to 20). Finally, the CQI was ranked into tertiles (Ts), a modification of an earlier approach [33–36].

### Anthropometric assessment

We measured the weight to the nearest 100 g using a Seca weighing scale (Seca GmbH and Co. KG; 22,089 Hamburg, Germany; Model: 8741321009; designed in Germany; made in China). The height measurements were carried out using the United Nations Children Emergency Fund height board. The height values were recorded to the nearest 0.1 cm. Body mass index (BMI) was determined by dividing the weight (in kilograms) by the height (in meters squared) [51]. The WC was obtained according to the World Health Organization standards. The values were measured at the mid-point between the lower border of the rib cage and the iliac crest using a non-stretchable fiber-glass measuring tape [52]. The WC was recorded to the nearest 0.1 cm.

### Assessment of demographic and lifestyle factors

All the data about the demographic and lifestyle factors (age, educational status, marital status, occupation, household size, household assets, parity, and physical activity) were collected from face-to-face interviews using structured questionnaires. To enable us to estimate the wealth status of each respondent’s household, we created a composite score for the list of household items enumerated. A higher score was a proxy for a higher wealth status [34]. The physical activity levels of the subjects were estimated using the International Physical Activity Questionnaire short form [53]. The questionnaire estimated the time and the number of days within the previous week each person spent on walking and on doing moderate-intensity and vigorous-intensity activities. The total physical activity in metabolic equivalent (MET) was calculated from the summation of the total walking, total moderate and total vigorous activities in MET-minutes/week score units.

### Statistical analysis

To ensure the compliance of data suitability with the chosen analytic techniques, the Kolmogorov–Smirnov test was used to evaluate the normality of the data. All study variables that failed the normality test were log-transformed before they were used in the regression models.

The general characteristics of subjects were presented according to MetS status as means ± standard deviations (SDs) for all continuous variables which met the normality test. For these variables, the independent t-test was used to assess the statistical significance. Also, we calculated the medians and interquartile ranges for variables that failed the normality test. To assess the statistical significance of those variables, we used the *Mann-Whitney test. Moreover,* percentages and simple counts for categorical variables were computed using the chi-square test*.*

*The sex-specific* energy-adjusted dietary CQI was used to classify controls into tertiles (Ts). To assess the differences of characteristics of controls across the Ts of CQI, the one-way analysis of variance (ANOVA) was used for continuous variables that the met normality test. We also calculated medians and interquartile ranges for those that failed the normality test.

The beta (β) coefficients and standard errors (SE) for linear regressions between sex-specific energy-adjusted CQI and individual components of the MetS were calculated. Additionally, the binary logistic regression was used to determine odds ratios (ORs) and 95% confidence intervals (CIs) for MetS across the Ts of the sex-specific energy-adjusted CQI. In model 1, we adjusted for age, sex and T2DM duration (years). In model 2, we further adjusted for energy, physical activity, BMI and educational status. These adjusted variables were variables that either showed a significant relationship with CQI or have the tendency to influence the relationship between CQI and MetS. All statistical analyses were done using IBM Statistical Package for Social Sciences (version 24; SPSS Inc.), and *p* <  0.05 was considered statistically significant.

## Results

Characteristics of the controls and cases are presented in Table 1. Total CHO intake was higher in the case group compared with the controls (*P* <  0.001). However, participants in the case group reported lower intake of total fiber (*P* < 0.001), WGTGR (*P* = 0.02), and CQI (*P* < 0.001) compared with participants in the control group. Overall, body weight, BMI, WC, systolic blood pressure, diastolic blood pressure, and TG were significantly higher (*P* < 0.001) amongst cases when compared with their control counterparts.

**Table 1:** Characteristics of T2DM patients without (controls) and with (cases) MetS

Variables	Controls	Cases	*P*-value
	*(n = 62)*	*(n = 62)*	
Age (years	45 [17.3]	47.5 [21.0]	0.9^‡^
**Sex, n (%)**			
Male	31 (50.0)	31 (50.0)	1.0^*^
female	31(50.0)	31 (50.0)	
**Education**			
Non-tertiary	42 (46.2)	49 (53.8)	0.2^*^
Tertiary	20 (60.6)	13 (39.4)	
**Occupation**			
Farming and trading	42 (45.2)	51 (54.8)	0.1^*^
Salary worker	20 (64.5)	11 (35.5)	
**Marital Status**			
Single	3 (33.3)	6 (66.7)	0.4^*^
Married	59 (51.3)	56 (48.7)	
^π^Medication use			
No	3 (42.9)	4 (57.1)	0.7^*^
Yes	59 (50.4)	58 (49.6)	
Physical activity (MET-minutes/week)	2260.5[4504.5]	2646.0[5475.3]	0.8^‡^
^¥^ Asset score	22.0 [4.3]	23.0 [6.2]	0.4^‡^
Duration of T2DM (years)	5.0 [6.0]	4.0 [4.0]	0.2^‡^
Household size	6.0 [4.0]	6.0 [5.0]	0.1^‡^
Energy intake (kcal/d)	148 1.8 ± 457.1	1756.90 ± 380.3	< 0.001^**^
Fat intake (g/d)	14.1 [6.2]	13.2 [8.4]	0.4^‡^
Protein intake (g/d)	40.5 [15.5]	42.6 [21.5]	0.1^‡^
CHO intake (g/d)	291.50 ± 103.4	358.9 ± 88.2	< 0.001^**^
Fiber intake (g/d)	20.5 [8.5]	13.6 [5.2]	< 0.001^‡^
RSCTC intake	0.89 [0.20]	0.88 [0.19]	0.3^‡^
WGTGR intake	0.53 [0.38]	0.20 [0.27]	< 0.001^‡^
Energy adjusted glycemic index	66.6 [3.2]	67.29 [3.3]	0.02^‡^
Energy adjusted glycemic load	212.9 [22.2]	220.2 [28.8]	0.050^‡^
CQI	15.0 [4.0]	10.0 [4.0]	< 0.001^‡^
Weight (Kg)	69.8 ± 13.1	82.7 ± 14.8	< 0.001^**^
Height (cm)	162.5 [13.5]	161.1 [12.03]	0.5^‡^
Body mass index (kgm^− 2^)	25.7 [7.4]	30.6 [7.7]	< 0.001^‡^
Waist circumference (cm)	81.8 [15.05]	114.0 [18.8]	< 0.001^‡^
Systolic blood Pressure (mmHg)	121.5 [6.0]	137.0 [23.5]	< 0.001^‡^
Diastolic blood pressure (mmHg)	76.5[11.2]	85.5 [12.0]	< 0.001^‡^
Triglycerides (mmol/l)	1.50 ± 0.39	2.18 ± 0.40	< 0.001^**^
HDL-C (mmol/l)	1.14 [0.26]	1.17 [0.25]	0.054^‡^
LDL-C (mmol/l)	3.33 [1.37]	3.31 [0.87]	0.7^‡^
TC (mmol/l)	5.19 [1.37]	5.54 [1.13]	0.053^‡^
^*Ω*^ Fasting blood sugar (mmol/l)	8.4 ± 2.5	9.0 ± 3.6	0.2^**^
*LDL-C/HDL-C ratio*	3.15[1.12]	2.72[1.37]	0.8^‡^

Data expressed as number (%) or mean ± standard deviation and median [interquartile range]; * *P-*value is for Chi-square; ** *P-*value is for independent t test; ‡ *P-value is for Mann-Whitney test.* ^π^metformin, glimepiride, atorvastatin, and/or amlodipine *usage.* ^*Ω*^ *Retrieved from patients’ folders.* ^¥^An ad hoc summary value was calculated from a list of household assets and room makeup. A score of 1 point was given for an item owned or room makeup includes local materials such as thatch or cow dung. As for the main source of cooking energy, drinking water and toilet facility, 1 point was given for unimproved source and 2 points awarded for improved sources. The total score range was from 37 to 74 with higher scores suggesting enhanced economic status. *Kcal/d* kilocalories per day; g/d: gram per day, *CHO* Carbohydrate, *WGTGR* ratio of CHO consumed from whole grains to CHO consumed from total grains, *RSCTC* ratio of CHO consumed from solid food items to total CHO consumed, *CQI* Carbohydrate quality index, *HDL-C* High density lipoprotein cholesterol, *LDL-C* Low density lipoprotein cholesterol. The cases and controls were matched on sex and age groups (18–39, 40–49, and 50–59 years)

Correlations between the sex-specific energy-adjusted CQI and fiber, RSCTC, WGTCR, and GI are presented in Table 2. After adjustment for age, sex and T2DM duration, education, BMI, energy and physical activity, a positive correlation was found between the sex-specific energy-adjusted CQI and fiber (*r =* 0.82; *P <* 0.001), RSCTC (*r =* 0.14; *P* = 0.1), and WGTCR (*r =* 0.78; *P <* 0.001). However, a negative correlation was found between dietary GI and the CQI (*r =* − 0.61; *P <* 0.001). Moreover, total fiber (25.3%), and RSCTC (28.9%) had a higher contribution to the overall CQI in females compared with males. However, in all subjects combined, total fiber (24.2%), WGTGR (24.4%), and GI (24.5%) had a similar contribution to the CQI. Meanwhile, the RSCTC (26.8%) showed the highest contribution to CQI (Table 3).

**Table 2:** Correlation between sex-specific energy-adjusted CQI and total dietary fiber, RSCTC, WGTGR, and GI^a^

CQI component	Partial Correlation (*n* = 124)	*P* value
	r	
Fiber	0.82	< 0.001
RSCTC	0.14	0.1
WGTGR	0.78	< 0.001
GI	−0.61	< 0.001

^a^Partial correlations between CQI and total dietary fiber, RSCTC, WGTGR, and GI were adjusted for age, sex and duration of type 2 diabetes, education, BMI, energy and physical activity. *CHO* Carbohydrate, *WGTGR* ratio of CHO consumed from whole grains to CHO consumed from total grains, *RSCTC* ratio of CHO consumed from solid food items to total CHO consumed, *CQI* Carbohydrate quality index

**Table 3:** Contribution of fiber, RSCTC, WGTCR, and GI to sex-specific energy-adjusted CQI intake (%)

	Fiber	RSCTC	WGTGR	GI
Male	*23.2*	24.7	25.7	26.5
Female	25.3	28.9	23.2	22.6
Total	24.2	26.8	24.4	24.5

*CHO* Carbohydrate, *WGTGR* ratio of CHO consumed from whole grains to CHO consumed from total grains, *RSCTC* ratio of CHO consumed from solid food items to total CHO consumed, *CQI* Carbohydrate quality index

Characteristics of 62 controls across sex-specific energy-adjusted CQI tertiles are presented in Table 4. Total dietary fat intake had a positive association with CQI (P for trend = 0.01). The CQI showed a positive relationship with total fiber (P for trend < 0.001), RSCTC (P for trend < 0.001), and WGTGR (P for trend < 0.001). Conversely, a negative association was found between the CQI and GI (P for trend = 0.001), and GL (P for trend = 0.04).

**Table 4:** Characteristics of 62 control group across tertiles of sex-specific energy-adjusted CQI

		CQI Tertiles (Ts)		
Range	T1 (7.0–12.0)	T2 (13.0–15.0)	T3 (16.0–20)	*P* for Trend
Number of participants	22	22	18	
CQI, median (g/d)	12.0	15.0	17.0	
Age (years), median [IQR]	45.0[20.8]	44.0[14.0]	49.0[19.0]	0.9
Sex				
Male	12(38.7)	9(29.0)	10(32.3)	0.4
Female	10(32.3)	13(41.9%)	8(25.8)	
Education				
Non-tertiary	13(31.0)	14(33.3)	15(35.7)	0.9
Tertiary	9(45.0)	8(40.0)	3(15.0)	
Occupation				
Farming and trading	14(33.3)	12(28.6)	16(38.1)	0.5
Salary worker	8(40.0)	10(50.0)	2(10.0)	
Duration of T2DM (years)	5.0[6.0]	4.5[3.5]	4.5[8.0]	0.3
Household size	6.0[3.3]	6.0[3.0]	5.5[4.3]	0.3
Asset score^¥^	22.0[5.3]	21.5[5.0]	21.5[6.5]	0.8
Physical activity (MET-minutes/week)	2439.0[4377.7]	2799.0[6255.0]	2113.5[4404.3]	0.8
Energy intake (kcal/d)	1465.8 ± 520.1	1467.9 ± 447.8	1518.15 ± 406.0	0.2
Fat intake (g/d)	12.3[5.2]	14.7[6.5]	16.96[6.07]	0.01
Protein intake (g/d)	38.4[12.1]	38.4[19.1]	46.06[20.03]	0.7
CHO intake (g/d)	291.3 ± 123.5	289.8 ± 94.1	293.7 ± 92.5	0.3
Fiber intake (g/d)	17.0[5.4]	21.3[6.5]	26.86[8.80]	< 0.001
RSCTC intake	0.85[0.21]	0.89[0.21]	0.90[0.11]	< 0.001
WGTGR intake	0.27[0.31]	0.55[0.37]	0.77[0.34]	< 0.001
Glycemic index	67.3[2.0]	66.6[2.5]	64.5[2.7]	0.001
Glycemic load	220.9[19.7]	212.0[17.3]	201.08[26.5]	0.04
Weight (Kg)	70.8 ± 14.8	67.7 ± 12.0	71.1 ± 12.4	0.1
Height (cm)	161.9[14.0]	165.7[14.1]	159.2[13.9]	0.2
BMI (kgm^−2^)	25.1[8.2]	24.7[7.5]	28.2[4.9]	0.7
Waist circumference (cm)	89.8[18.3]	78.3[14.3]	85.1[15.5]	0.2
Systolic blood Pressure mmHg	123.5[4.5]	120.0[7.7]	122.0[7.8]	0.9
Diastolic blood pressure (mmHg)	76.0[13.2]	77.0[6.7]	72.5[12.8]	0.7
Triglycerides (mmol/l)	1.60 ± 0.35	1.39 ± 0.43	1.48 ± 0.33	0.2
HDL-C (mmol/l)	1.16[0.29]	1.07[0.29]	1.14[0.23]	0.8
LDL-C (mmol/l)	3.20[1.31]	3.51[1.55]	3.12[1.56]	0.2
Total cholesterol (mmol/l)	5.17[1.22]	5.27[1.65]	4.97[3.76]	0.3
^*Ω*^ Fasting blood sugar (mmol/l)	8.53 ± 2.70	8.70 ± 2.44	7.71 ± 2.27	0.5
*LDL/HDL ratio*	2.83[1.22]	3.38[1.14]	3.07[1.04]	0.4

Data expressed as number (%) or mean ± standard deviation and median [interquartile range]; ^*Ω*^ *Retrieved from patients’ folders.*^¥^An ad hoc summary value was calculated from a list of household assets and room makeup. A score of 1 point was given for an item owned or room makeup includes local materials such as thatch or cow dung. As for the main source of cooking energy, drinking water and toilet facility, 1 point was given for unimproved source and 2 points awarded for improved sources. The total score range was from 37 to 74 with higher scores suggesting enhanced economic status. *Kcal/d* kilocalories per day, *g/d* gram per day, *CHO* Carbohydrate, *WGTGR* ratio of CHO consumed from whole grains to CHO consumed from total grains, *RSCTC* ratio of CHO consumed from solid food items to total CHO consumed, *CQI* Carbohydrate quality index, *HDL-C* High density lipoprotein cholesterol, *LDL-C* Low density lipoprotein cholesterol, P for trend test was performed using linear regression models where categorical covariates were entered as continuous variables using their dummies. Also, the CQI tertile classes were replaced by their respective medians

The relationship between the sex-specific energy-adjusted CQI and anthropometric variables, FBS, and serum lipid concentrations are presented in Table 5. After an adjustment for the effects of age, sex and T2DM duration, the CQI was negatively associated with waist circumference (WC) (β = − 17.96; SE = 4.35; P for trend < 0.001), systolic blood pressure (SBP) (β = 16.09; SE = 4.74; P for trend < 0.001), diastolic blood pressure (DBP) (β = − 7.62; SE =2.97; P for trend = 0.007) and TG (β = − 0.45; SE = 0.12; P for trend < 0.001). However, the CQI showed a positive association with HDL-C (β = 0.23; SE =0.20; P for trend = 0.009). After additional adjustments for the effects of education, energy, physical activity, and BMI (**except when it was used as a dependent variable**), WC (β = − 17.29; SE =4.00; P for trend < 0.001), SBP (β = − 15.74; SE = 4.69; P for trend < 0.001), DBP (β = − 7.23; SE =2.97; P for trend =0.02) and TG (β = − 0.43; SE = 0.11; P for trend < 0.001) still had a negative association with the CQI. Also, a significant positive relationship between the CQI and HDL-C (β = 0.24; SE =0.20; P for trend =0.01) was still maintained. The CQI showed a positive association with the LDL-C/HDL-C ratio (β = 0.14; SE =0.20; P for trend =0.04).

**Table 5:** Linear regression analysis for cardiovascular risk factors across tertile (T) categories of sex-specific energy-adjusted CQI^a^ (*n =* 124)

	CQI			
	T1 (7.0–12.0)	T2 (13.0–15.0)	T3 (16.0–20)	*P* for Trend
No. of cases/controls	53/22	6/22	3/18	
CQI, median (g/d)	12.0	15.0	17.0	
Model 1				
Body mass index (kg/m^2^)	Reference^b^	−4.25 (1.67)	−1.06 (1.87)	0.1
Waist circumference (cm)	Reference	−17.09 (3.88)	−17.96 (4.35)	< 0.001
Systolic blood pressure (mm Hg)	Reference	−9.86 (4.22)	−16.09 (4.74)	< 0.001
Diastolic blood pressure (mm Hg)	Reference	−4.22 (2.65)	−7.62 (2.97)	0.007
Blood glucose (mmol/l)	Reference	0.02 (0.68)	−1.16 (0.77)	0.2
Total Cholesterol (mmol/l)	Reference	−0.12 (0.22)	−0.13 (0.24)	0.5
Serum triglycerides (mmol/l)	Reference	−0.41 (0.11)	−0.45 (0.12)	< 0.001
HDL-C (mmol/l)	Reference	0.46 (0.18)	0.23 (0.20)	0.009
LDL-C (mmol/l)	Reference	− 0.14 (0.04)	− 0.08 (0.05)	0.3
LDL-C/HDL-C	Reference	0.21 (0.18)	0.14 (0.20)	0.050
**Model 2**				
Body mass index (kg/m^2^)	Reference	−3.75 (1.55)	−0.71 (1.73)	0.2
Waist circumference (cm)	Reference	−13.43 (3.67)	−17.29 (4.00)	< 0.001
Systolic blood pressure (mm Hg)	Reference	−7.46 (4.31)	−15.74 (4.69)	0.001
Diastolic blood pressure (mm Hg)	Reference	−3.03 (2.73)	−7.23 (2.97)	0.02
Blood glucose (mmol/l)	Reference	0.01 (0.72)	−1.18 (0.79)	0.2
Serum triglycerides (mmol/l)	Reference	−0.31 (0.10)	−0.43 (0.11)	< 0.001
HDL-C (mmol/l)	Reference	0.55 (0.19)	0.24 (0.20)	0.01
LDL-C (mmol/l)	Reference	−0.15 (0.04)	−0.07 (0.05)	0.2
LDL-C/HDL-C	Reference	0.28 (0.19)	0.14 (0.20)	0.04

^a^Data presented as β (SE)

β: unstandardized coefficient; SE: standard error (SE)

^b^T1 considered as reference and other tertiles compared with this tertile

*CQI* Carbohydrate quality index, *HDL-C* High density lipoprotein cholesterol, *LDL-C* Low density lipoprotein cholesterol. CQI was categorized into tertiles according to the distribution of the control group. For overall subjects, the tertile classes for CQI were: T1(4.0–6.0); T2 (7.0–8.0); T3 (9.0–12.0); *OR* Odds ratios, *CI* Confidence interval

*OR* Odds ratio, *CI* Confidence interval; Model 1: Adjusted for age, sex and duration of type 2 diabetes; Model 2: Further adjusted for education, energy, physical activity, and body mass index (**except when it was used as a dependent variable**). To test for a trend across the CQI, the median for each tertile of CQI was used as a continuous variable

Unadjusted and adjusted ORs and 95% CIs for MetS across Ts of CQI are provided in Table 6. After an initial adjustment for the effects of age, sex and T2DM duration, the CQI had an inverse relationship with the odds of MetS (OR_tertile 3 vs.1_ 0.07; 95% CI 0.02–0.26; p-trend < 0.001), however, this was a weak relationship though it attained a statistical significance. Moreover, after additional adjustment for BMI, energy, physical activity, and education, there was still an inverse association between the CQI and the odds of MetS (OR_tertile 3 vs.1_ 0.05; 95% CI 0.01–0.23; p-trend < 0.001).

**Table 6:** Odds ratios (ORs) and 95% confidence intervals (CIs) MetS according to tertiles (Ts) of sex-specific energy-adjusted CQI indices

CQI	T1	T2	T3	*P* for Trend
No. of cases/controls	53/22	6/22	3/18	
Median	12.0	15.0	17.0	
Range	(7.0–12.0)	(13.0–15.0)	(16.0–20)	
OR (95% CI)				
Model 1	1.00 (Ref)	0.11(0.04–0.32)	0.07(0.02–0.26)	< 0.001
p		< 0.001	< 0.001	
Model 2	1.00 (Ref)	0.13(0.04–0.42)	0.05(0.01–0.23)	< 0.001
p		0.001	< 0.001	

CQI, PSR, ∑**ω-3/**∑**ω-6**, h/H, AI, TI and LI were categorized into tertiles according to the distribution of the control group

For overall subjects, [CQI: T1(6.0–10.0); T2 (11.0–14.0); T3 (15.0–20.0)

*OR* Odds ratios, *CI* Confidence interval, *CQI* Carbohydrate quality index

Model 1: Adjusted for age, sex and duration of type 2 diabetes; Model 2: Further adjusted for BMI, energy, physical activity, education

To test for a trend across the sex-specific energy-adjusted CQI, the median for each tertile was used as a continuous variable

## Discussion

The present study examined the relationship between a dietary sex-specific energy-adjusted CQI and the odds of MetS among T2DM subjects. There was an inverse association between the CQI and the odds of MetS. Also, a positive correlation was found between the CQI and fiber, RSCTC, and WGTCR but the CQI showed a negative relationship with GI. Moreover, the CQI had a positive association with high HDL-C, and an inverse relationship with WC, SBP, DBP, and TG.

Previous studies among T2DM subjects in Sub-Saharan Africa only focused on assessing lifestyle factors such as the level of physical activity in Nigeria [38], and in Ghana, barriers to adequate nutrition [39, 40] and associations between dietary diversity and BMI [54]. In the country, the majority of studies among T2DM subjects were focused on assessing the prevalence of MetS but did not investigate the possible dietary risk factors associated with MetS prevalence [3–7]. This perspective only provides limited insight into how to develop diet-related interventions for the prevention of metabolic disorders. Therefore, the present comprehensive evaluation of the quality of dietary carbohydrates consumed among Ghanaian T2DM subjects may help refine nutrition policy in the country, especially that about 90% of the daily energy need in the Ghanaian adult population comes from dietary carbohydrates [41].

In our study, the CQI was negatively associated with WC, SBP, DBP, TG, and the odds of MetS. However, the CQI showed a positive association with HDL-C. These associations as observed in the present study contravened previous results reported from a cross-sectional study in South Koreans, in which CQI failed to show a relationship with MetS and its component factors the WC, HDL-C, TG, TC, and FBS [35]. Several factors may have contributed to the variations in the results of the two studies. For instance, we studied T2DM subjects in a case-control setting whereas, they analyzed data on a mixed population of adults with some far older than the age limit set for our study. Moreover, a repeated 24-h dietary recall was used in the present study whilst they used a single 24-h recall. Furthermore, we used the residual method to adjust for energy intake before the calculation of the sex-specific energy-adjusted CQI whereas, they used unadjusted dietary fiber, GI and carbohydrates in the CQI calculation.

Notwithstanding the variations in results between the present study and the Korean study [35], the two studies share some similarities. In line with the inverse association between the CQI and the odds of the measures of hypertension (SBP and DBP) found in the present study, the Korean study also reported an inverse association between CQI and prevalence of hypertension. Also, the inverse association between the CQI and the odds of high WC agrees with our previous results reported in Ghanaian healthy women, in which CQI showed a negative association with abdominal obesity indicated by WC. Diets high in CQI are healthy since a higher CQI is directly associated with fiber intake, whole grain product consumption, solid carbohydrate food consumption, and are low in GI. In numerous previous studies, these components of the CQI have shown favorable relationships with MetS and/or insulin sensitivity, high HDL-C, and low TG, LDL, SBP, DBP, and WC in T2DM subjects and the general population [22, 26–28, 30, 55–58].

Although the mechanisms that underlie the inverse association between the CQI and the MetS in the present study are unknown, previous mechanistic studies have espoused various explanations on how the components of the CQI may affect the occurrence of MetS and its risk factors. For example, dietary fiber is associated with lower postprandial glucose levels and increased insulin sensitivity in diabetics and healthy subjects [59–61], thereby preventing the insulin resistance component of the MetS. Also, the results from clinical trials [62–70] and meta-analyses [71, 72] support the cholesterol-reducing effects and the prevention of hypertension and the improvements in the clinical features of MetS (glycemic control, lipoprotein profile, BMI, and blood pressure). Also, whole-grain consumption has the effect of prolonged satiety and also the capacity to slow down starch digestion and absorption and may as well lead to lower glucose and insulin responses [73, 74], and the prevention of obesity [75, 76], which are beneficial conditions for reducing the development of MetS. On the other hand, there are evidences from randomized controlled clinical trials and meta-analysis of randomized controlled trials that in adults with MetS, low GI diets can cause significant decreases in anthropometric measurements, blood pressure, FBS and serum lipid profiles (TG, TC, LDL-C, HDL-C) [22, 55–58], which are beneficial for the prevention of MetS development. Moreover, liquid CHO diets such as the sugar-sweetened beverages are often high in GI [44, 77]; can increase postprandial blood glucose levels, decrease insulin sensitivity and increase the risk of obesity and overweight [77], which are also important risk factors for the MetS occurrence.

The present study has a number of important strengths. As far as we are aware, this study was the first to investigate CQI taking into account the sex-specific difference in carbohydrate consumption and its relationship with the MetS in Africa. Moreover, necessary precautions were taken to enhance data quality and study results during both data collection and analysis. However, the study was not without some inadequacies. First, the sample size was very small which may have lacked the necessary statistical power to reveal very important relations between the CQI and the MetS and/or its components. Second, this was an observational study, the findings did not establish a causal relationship between CQI and MetS. Third, we used 24-h dietary recall for dietary intake assessment therefore, there was the chance that some participants may have misreported their dietary intake due to memory-related issues. Additionally, there was the likelihood of underreporting of foods perceived to be undesirable for health and the chance for over-reporting for food items perceived to be healthy. Although we controlled for some potential confounders, we cannot discount the effects of seasonal variations in food intake, genetic impact, and residual confounding. With these potential errors, the results ought to be interpreted with caution.

## Conclusions

The present study found an inverse association between the CQI and the odds for MetS and reductions in WC, SBP, DBP, and TG but an in increase in HDL-C. This finding provides a piece of useful information which may guide carbohydrate nutrition planning for MetS prevention, particularly among T2DM subjects. However, experimental studies are needed to establish a possible causal relationship between the odds for MetS and dietary CQI intake.

## Data Availability

The datasets used to support the findings of the study are available from the corresponding author upon request.

## Abbreviations

CQI: Carbohydrate quality index
GI: Glycemic Index (GI)
GL: Glycemic load
CHO: Carbohydrates
WGTGR: Ratio of CHO consumed from whole grains to CHO consumed from total grains
RSCTC: Ratio of CHO consumed from solid carbohydrate food items to total CHO consumed
BMI: Body mass index
WC: Circumference
IDF: International Diabetes Federation
UNICEF: United Nations Children Emergency Fund
WHO: World Health Organization
MetS: Metabolic syndrome
T2DM: Type 2 diabetes mellitus
FBS: Fasting blood sugar
TG: Triglycerides
TC: Total cholesterol
LDL-C: Low-density lipoprotein cholesterol
HDL-C: High-density lipoprotein cholesterol
BP: Blood pressure
MET: Metabolic equivalent
SDs: Means ± standard deviations
ANOVA: One-way analysis of variance
CI: Confidence intervals
β: Unstandardized beta coefficient
SE: Standard error of beta
OR: Odds ratio
P: Statistical significance
Q: Quintile
T: Tertile
